# Tumor hypoxia and role of hypoxia-inducible factor in oral cancer

**DOI:** 10.1186/s12957-023-03284-3

**Published:** 2024-01-11

**Authors:** Pooja Singh, Monika Rajput, Manoj Pandey

**Affiliations:** Department of Surgical Oncology, Institute of Medical Sciences, Banaras Hindu University, Varanasi, 221005 India

**Keywords:** Oral cancer, Hypoxia, TP53, VEGF, Glucose metabolism, Bevacizumab, Pazopanib, Sunitinib, Axitinib

## Abstract

**Background:**

Head and neck cancer (HNC) is one of the most frequent malignancies in Asian males with a poor prognosis. Apart from well-known prognostic indicators, markers of tumor hypoxia can help us predict response to treatment and survival.

**Methods:**

A review of the literature on the present evidence and potential clinical importance of tumor hypoxia in head and neck cancer was carried out. The data obtained from the literature search is presented as a narrative review.

**Results:**

The literature shows possible associations between prognosis and low tumor oxygenation. Intermediate hypoxia biomarkers like HIF-1, GLUT-1, miRNA, and lactate, can help in predicting the response to therapy and survival as their altered expression is related to prognosis.

**Conclusions:**

Hypoxia is common in HNC and can be detected by use of biomarkers. The tumors that show expression of hypoxia biomarkers have poor prognosis except for patients with human papilloma virus-associated or VHL-associated cancers. Therapeutic targeting of hypoxia is emerging; however, it is still in its nascent stage, with increasing clinical trials hypoxia is set to emerge as an attractive therapeutic target in HNC.

## Introduction

Head and neck cancer (HNC) is a formidable oncological challenge. HNC comprises a diverse group of malignancies affecting the oral cavity, pharynx, pyriform sinus, larynx, and adjacent structures. Despite numerous advances in diagnosis and introduction of newer therapeutic modalities, the prognosis for HNC patients remains suboptimal, underscoring the need to unravel the complex molecular underpinnings of this aggressive disease [1].

In recent years, increasing consideration has been given to understand the role of the tumor microenvironment in shaping cancer behavior and treatment response [2]. Among the various factors influencing tumor progression, hypoxia has emerged as a pivotal player in driving the pathobiology of HNC. Tumor hypoxia is defined as non-physiological low oxygen tension in the tumor relative to the surrounding tissue. Regardless of size or histology, more than half of all solid tumors show heterogeneous regions of hypoxia. A functional definition may be that the “tumor hypoxia starts when hypoxia-inducible factor (HIF) subunits become stabilized due to limited oxygen availability compared to oxygen demand” [3, 4]. Factors like physical pressure of oxygen, utilization of oxygen by cells, perfusion and diffusion, angiogenesis, and the distance of vessels from the tissue as in the case of edema can determine the hypoxia. Systemic diseases like anemia and chronic obstructive or restrictive lung disease can also influence oxygenation and thus hypoxia.

This comprehensive research review aims to dissect the multifaceted role of hypoxia in the context of HNC. By exploring the hypoxia biomarkers like HIF-1, GLUT-1, miRNA, and lactate, we seek to unravel the mechanisms through which hypoxia shapes the aggressive phenotype of HNC. Additionally, this review delves into the potential implications of hypoxia on therapeutic resistance, prognosis, and overall treatment outcomes [5].

The review will also discuss the implications of hypoxia in different subtypes of HNC, particularly focusing on human papillomavirus (HPV)-associated or von Hippel-Lindau (VHL)-associated cancers, which show different prognostic patterns despite expressing hypoxia biomarkers. The potential therapeutic targeting of hypoxia pathways and its emerging status as an attractive therapeutic approach will also be examined [2].

## Methods

A PubMed search was performed using the keywords tumor hypoxia, nitric oxide, and intermediate biomarkers like HIF-1 alpha, CA-9, HIF1 beta, VEGF, lactate; other factors like smoking, anemia, and miRNA and head and neck cancer; or head and neck squamous cell carcinoma. Articles discussing the mechanism of hypoxia in HNC, treatment strategies, biomarker, and prognosis were included. Cell line studies and reviews were excluded.

## Results

A total of 161 articles were found, of which 75 were found to be relevant after exclusion. Understanding the association between tumor hypoxia and clinical outcomes in head and neck cancer is highly relevant for guiding treatment decisions and improving personalized therapeutic approaches. However, there is a research gap in the comprehensive evaluation of intermediate hypoxia biomarkers and their clinical significance in HNC [6]. Articles were reviewed for clinical and scientific evidence and were divided by subtopics that are used as subheadings and discussed.

### General conditions influencing tumor hypoxia

#### Anemia

Anemia plays an important role in tumor hypoxia; it is defined as a “hemoglobin (Hb) level less than 12.0 g/dl in females and less than 13.8 g/dl in males.” Acute anemia occurs when the RBC count falls abruptly, most commonly due to hemolysis or acute bleeding. Chronic anemia, on the other hand, is generally a gradual decline in erythrocytes, and the causes include iron or other nutritional deficiencies, chronic disease, drug-induced, and others. In oral cancer, the association between anemia and tumor hypoxia is well documented. Anemia can aggravate hypoxia in the tumor microenvironment, contributing to increased tumor growth and therapeutic resistance. Furthermore, hypoxia is linked to a higher risk of regional lymph node metastasis, and hence, the importance of anemia and tumor hypoxia in clinical outcomes in oral cancer cannot be over-emphasized. Hb greater than 12 g/dl is reported as an independent prognostic factor [7–9]. Anemia impairs tissue and tumor oxygenation leading to hypoxia which in turn reduces the efficacy of radiotherapy and chemotherapy as they depend on the production of nascent oxygen to produce maximum response [10–12]. It has been reported that Hb levels between 12 and 14 g/dl are optimal for tumor oxygenation even though there is no correlation between Hb level and pO_2_ [13].

Contrary to the above, some of the researchers suggest that a blood transfusion leads to a worsening of the prognosis [14, 15] or at least the blood transfusion has no effect on the survival of HNC patients before or after radiation. This contradiction is explained by hypothesizing that the endothelial growth factors may leak from aging red blood cells, thus promoting the growth of tumors and negatively affecting immunological control.

Erythropoietin (EPO), a glycoprotein, regulates erythrocyte production. Erythropoietin and its receptor are found to be expressed in 95% of HNC. A positive correlation between erythropoietin and erythropoietin receptor expression, HIF-1alpha, and CA-9 has been reported. However, no correlation of erythropoietin or its receptors with Hb was observed [16]. As the optimal results are obtained between Hb levels of 12–14gm/dL, this could explain why EPO fails to improve the outcome as the Hb often rises above the optimum level and this leads to increased resistance to the flow of blood. Except for cervical cancer, no association has been found between the Hb levels and the intermediate markers like CA-9, HIF 1, and 2 in other cancers including HNC [12]. The Hb levels alone cannot be used as a surrogate for oxygen levels in the tissue as other factors like blood flow, Hb saturation, and dissociation can also contribute to hypoxia in patients [11].

The importance of hemoglobin levels as a prognostic indicator provides compelling evidence for the need to treat anemia in HNC patients to reverse tumor hypoxia; however, the evidence suggests that optimal levels between 12 and 14 gm/dL be maintained.

### Smoking

Smoking is one of the main risk factors and the source of carbon monoxide (CO) in patients with HNC. CO has a very high affinity to bind with Hb, higher than O_2_, and this leads to the formation of carboxyhemoglobin (HbCO) [17]. Formation of HbCO leads to the dissociation of oxygen and reduced oxygenation of the tissues producing hypoxia-like condition and stimulating a hypoxia cascade. It is reported that the level of HbCO in smokers can rise to 12% (compared to 4.6% in non-smokers) and can lead to a 25–50% reduction in O_2_ available to the tumor [17, 18].

Furthermore, CO itself can stimulate the expression of hypoxia-inducible factor-1 alpha (HIF-1), a transcription factor involved in the cellular response to hypoxia. HIF-1 controls the production of genes involved in angiogenesis, glucose metabolism, and apoptosis, and its overexpression leads to tumor growth and therapeutic resistance. As a result, the link between CO and blood flow in smoking may lead to tumor hypoxia and a poor prognosis.

### Alcohol

Apart from smoking consumption of alcohol or alcohol alone or combined with smoking is a risk factor for HNC. Alcohol may potentiate the effect of smoking by acting as a solvent and thereby when consumed together has a multiplicative effect on carcinogenesis. Till date, studies have failed to establish a direct relationship between alcohol with hypoxia; however, indirect evidence points to activation of HIF-1 via oxidative stress as seen in the liver in animal models [19, 20]. Acetaldehyde, a known carcinogen, is produced in the liver and mucosa by alcohol dehydrogenase (ADH). ADH can directly cause DNA damage while alcohol facilitates its entry into the mucosa by altering the physiology [21]. Despite the fact that there is no direct evidence of alcohol-producing hypoxia, but may do so by its metabolites [22] or its effect on lipid metabolism [23], and hence, it can activate hypoxia cascade through HIF 1 and 2 or HIF independent mechanism.

### Human papillomavirus (HPV)

The relationship of HPV with hypoxia is controversial as the results of studies are contradictory. HPV-16 has been shown to induce HIF-1 alpha expression in HNC [24, 25] while other studies failed to find any relationship. However, it has been shown that patients who are HPV-negative and HIF-1 alpha-positive have the worst prognosis as though HPV-positive tumors are fast growing, they have excellent response to treatment [26].

**Fig. 1:**
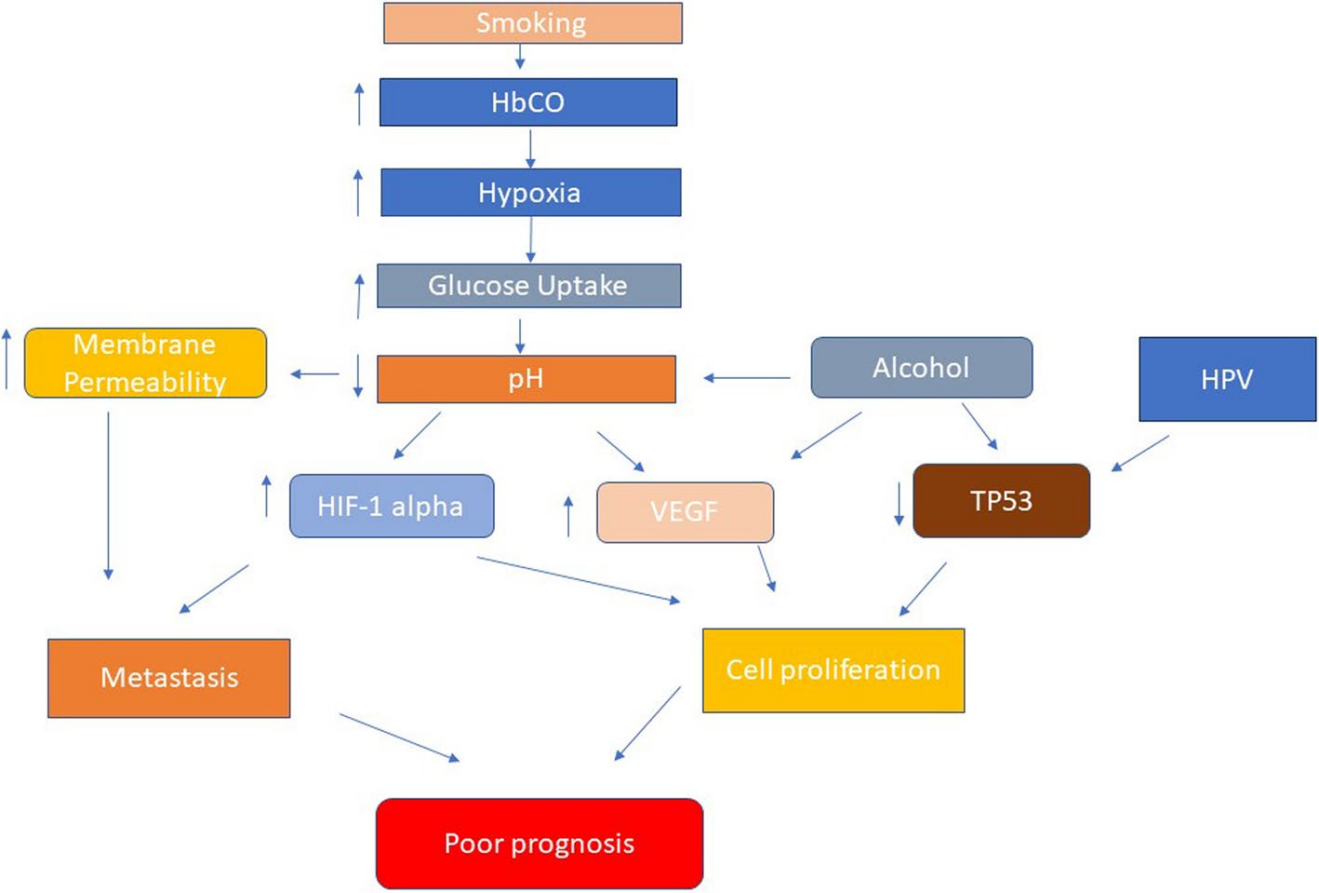
Hypoxia pathway in the presence of smoking, alcohol and HPV in HNSCC (Modified from Bredell MG, Ernst J, El-Kochairi I, Dahlem Y, Ikenberg K, Schumann DM. Current relevance of hypoxia in head and neck cancer. Oncotarget. 2016 Aug 2;7(31):50781-50804. doi: 10.18632/oncotarget.9549. PMID: 27434126; PMCID: PMC5226620. Open access CC By 4.0)

Various studies on hypoxia and HPV have shown an inverse relation with angiogenic factors like high angiogenic factors in the presence of HPV [27], higher rates of oxidative phosphorylation in HPV-positive, and high glycolysis in HPV-negative tumors [28]. Impaired DNA repair mechanism in the presence of HPV while intact repair in the absence of HPV [29]; a higher rate of immune cells in positive and reduced immunogenicity in the absence of HPV [30]; and p53 suppression in the presence of HPV while mutations in absence of it, besides other established mechanisms of hypoxia-like HIF-1, GLUT 1, and CA-9. The pathway of hypoxia induced by tobacco smoking, alcohol, and HPV is given in Fig. 1.

#### Immune system

Cigarette smoke has also been implicated to work through modulating the immune system and inflammation in patients with oral cancer [31]. Hypoxia has been shown to influence the immune defense mechanism in cancer by promoting the growth of regulatory T cells and myeloid-derived suppressor cells, both of which have been found to reduce antitumor immunity, is one such mechanism. Hypoxia can also alter the phenotypic and function of dendritic cells and macrophages, leading to reduce antigen presentation and cytokine production. Tumor cells can create immunological checkpoint molecules such as PD-L1 and CTLA-4 in response to hypoxia, which limits T cell activation and proliferation. Hypoxia can also activate signaling pathways that can promote tumor growth and survival while inhibiting immune response by boosting immunosuppressive molecules and lowering immune-activating molecules [32].

#### Biomarkers of hypoxia

In the presence of hypoxia, cells exhibit changes, increased angiogenesis and altered cell growth, and increased survival. This response is mainly brought about through HIF [33].

### Hypoxia-inducible factor (HIF)

HIF 1-alpha is the most widely characterized dimeric protein expressed in the cytoplasm with alpha and beta subunits [34] with a very short half-life. Under normoxic conditions, HIF1-alpha is expressed, along with prolyl hydroxylase (PHDs), that binds to HIF1-alpha leading to hydroxylation of 2 proline residues and acetylation of lysine residue. This increases the affinity of HIF-1 alpha for the von-Hippel Lindau gene (pVHL) product, leading to its proteasomal degradation [35]. As a consequence, in the presence of oxygen, HIF1-alpha is rapidly degraded and hence deactivated.

Hypoxia reduces the activity of PHDs, and consequently, HIF1-alpha translocates to the nucleus. Stabilization of HIF-1 alpha takes place by dimerizing with HIF1-beta, leading to activation of hypoxia cascade and activation of genes involved in maintaining oxygen homeostasis [36]. Recently, HIF-1α, C1772T and G1790A polymorphisms have been found to be associated with HNC [37]. In HNC cell lines HIF-2 alpha has been found to act through epidermal growth factor receptors, activating downstream signaling pathway [38]. It also alters the expression of VEGF, EPO, CA-9, glucose transporter (GLUT-1), and plasminogen activator inhibitor-1 (PAI-1). While expression of HIF-1 is controlled by nitric oxide [39, 40]. It is also controlled by cytokines and growth factors such as TGF-beta [41], reactive oxygen species, and insulin [42] besides hypoxia.

The expression of HIF 1 alpha is an early event in carcinogenesis [43]. HIF-1a is expressed in nearly 30% of tumors, while HIF-2alpha is expressed in 14%. A strong correlation exists between the expression of HIF-1 and 2. Other than hypoxia use of tobacco and alcohol has also been shown to influence expression of HIF [44, 45].

Generally, HIF-1 alpha expression is associated with poor prognosis in cancer patients [46]. In tongue cancer, HIF 1alpha expression is found to be an independent prognostic factor [47]. Coexpression with CA-9 has been reported to have poor outcomes in HNC [48]. However, HIF-1 and HIF-2 alpha expression by immunochemistry in patients undergoing surgery for HNC has been found to be associated with better disease-free and overall survival [49]. A higher 5-year survival and longer disease-free survival is reported by others [50, 51]. These results suggest that the effect of HIF expression on cancer survival is not clear and may depend on co-expression, molecular subtypes, and the therapies chosen to treat these patients; further studies hopefully will clear the association.

### Glucose transporter 1 (GLUT1)

Glucose transporter 1 (GLUT1) is a membrane transporter of glucose encoded by the solute carrier family 2 (SLCO2A1). GLUT1 transports glucose across the cell membrane down its concentration gradient. HIF-1 regulates the glucose transporter switch to open or close by binding and dissolution to GLUT-1. Expression of GLUT 1 has been studied in HNC and its expression has not been found to be associated with age, gender, TNM stage, or subsite, though this was significantly higher than precancerous normal tissue [52]. A meta-analysis of biomarkers of hypoxia published in 2015 reported a single study evaluating outcomes in HNC using GLUT-1 as a marker and reported a significant lowering of hazard if GLUT-1 was expressed [53, 54].

### Vascular endothelial growth factor (VEGF)

VEGF is a hypoxia-responsive gene that plays a key role in the development of tumor neovascularization [55]. In HNC, increased VEGF in tumor cells is associated with poor prognosis. This poor prognosis is attributed to higher clinical and nodal stages and the presence of metastasis [56]. An inverse relationship is observed with oxygen concentration, wherein the lowing of oxygen leads to an increase in VEGF expression [57–59]. In EGFR-mutant lung cancer, hypoxia has been shown to activate the VEGF pathway and a dual blockade is being suggested as a promising therapeutic activity [60]. A combination of anti-angiogenic therapy with radiation has also been shown to improve response [61]. The result of anti-angiogenic therapy is contrary to the belief that producing tumor hypoxia leads to therapeutic resistance and poor prognosis; however, the results have been promising and suggest an alternate pathway of action that may be independent of hypoxia.

### TP53

TP53 gene encodes p53 are a tumor suppressor protein. TP53 is most commonly found to be mutated in HNC [62] and is associated with tobacco use. One of its numerous activators is oxidative stress. In gastric and oesophageal cancer, it is demonstrated that mutation of TP53 leads to hypoxia and activation of the hypoxia cascade [63]. However, both HIF-1 and TP53 failed to show any correlation with tumor hypoxia using F-fluoromisonidazole (F-FMISO) PET in HNC [64]. However, in p16-positive tumors, the PET findings correlate with hypoxia biomarkers [65]. These contrasting results in HPV-positive tumors suggest that the blockade of TP53 may have a different pathway than mutant TP53 [66]. It is proposed that in HPV-negative tumors the action of TP53 may not be through activation of hypoxia cascade. In a cohort of supraglottic laryngeal carcinoma, no correlation of HIF-1 was found with TP53 expression [67]. TP53 does show an association with hypoxia in HPV-positive tumors; however, no association is demonstrated in HPV-negative tumors. Hence, at present, it is debatable that TP53 is a biomarker for hypoxia, and it appears to be associated with smoking or alcohol use in HNC and may be a surrogate [68].

### MicroRNAs

MicroRNA(miRNAs) are short noncoding RNAs that post-transcriptionally regulate target messenger RNAs [69]. Chen et al. reported 7 miRNAs to be associated with hypoxia, of which 3; miR-223, miR-34b, and miR-210 were upregulated while 4, miR-100, miR-99a, miR-125b, and miR-375 were downregulated [70]. Extracellular vehicles containing miR 192 and 215 have also been implicated in HNC through hypoxia-induced fibroblast development [71].

miR-21 is the most researched miRNA that has been found to be upregulated in various cancers [72], in HNC, and it has been shown to induce cancer-associated fibroblast activation and is proposed as an attractive target [73]. The action is proposed to be mediated by inhibiting the expression of FIH protein as MiR21 directly binds to FIH mRNA preventing its transcription in HNC. miR-31 and miR-184 also exhibit similar modes of action and can inhibit epithelial-mesenchymal transition in HNC, thus preventing the tumor spread in the presence of hypoxia and improving prognosis [74].

miR-210 is induced by hypoxia in cells [75]. Its expression correlates with HIF-1 alpha, and CA-9 the biomarkers of tumor hypoxia in HNC [76] and other cancers like pancreatic cancer, [77] anaplastic thyroid cancer [78] miR210, in head neck paragangliomas associated with VHL gene mutation has been found to be activated along with HIF-1 [79]; however, it has been found to be independent of SDH mutation [80]. Other genes that have been found to be targeted by miR210 are PLK1, MCT1, and MCT4 [81, 82].

**Fig. 2:**
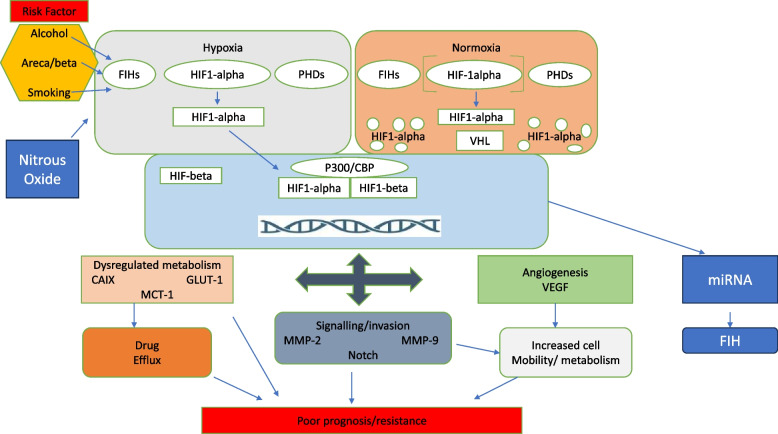
Causes and downstream pathway of hypoxia in HNSCC prognosis. (Modified and reproduced with permission from Kujan O, Shearston K, Farah CS. The role of hypoxia in oral cancer and potentially malignant disorders: a review. J Oral Pathol Med. 2017 Apr;46(4):246-252. doi: 10.1111/jop.12488. Epub 2016 Aug 25. PMID: 27560394

The research on miRNA and its association with hypoxia is still ongoing in HNC and several future developments are expected. Till date, miR 210 appears to be the most significant of all miRNAs found to be associated with hypoxia. The pathways of hypoxia and its effect on prognosis are detailed in Fig. 2.

### Hypoxia-targeted therapies

Generally, the hypoxia target therapies reported in literature either target HIF-1 or VEGF. HIF-directed therapies have focused on decreasing HIF-alpha mRNA, HIF protein synthesis, increasing its degradation or dimerization, or its transport across the nuclear membrane [83]. Trichostatin A (TSA), a histone deacetylase inhibitor, when tested in a cell line led to a decrease in cell proliferation and invasion besides reducing the basal level of HIF protein [84]. Glucosamine hydrochloride (GS-HCl) has also been found to reduce the proliferation of the HNC cell line [85]. PT2399, a selective HIF-2 antagonist has been found to suppress carcinogenesis in renal cell carcinoma (RCC) cell lines [86].

The approaches being utilized in clinical trials include blocking of transcription using GL331 [87], anthracyclines [88], steroids [89], topoisomerase inhibitors [90], microtubule binding agents [91], and aminoflavone [92]. The second approach is to reduce stability or inhibit dimerization of HIF using histone deacetylase (HDAC) inhibitors, drugs being tested include Panobinostat [93], MPT0G157 [94], Vorinostat [95], romidepsin [96], belinostat [97], and chidamide [98].

Targeting the PAS domains of HIF-1α and HIF-2α leads to inhibition of heterodimerization, and this is being tested using numerous compounds like PT2399, PT2977, acriflavine, Kaempferol, PD98059, and PT2385 [99]. Other drugs targeting multiple or differing mechanisms being tested are calcium channel blockers [100], PX-478 [101] bortezomib, a proteasome inhibitor [102].

### VEGF-targeted therapies

Anti-angiogenic agents have been tried for the treatment of oral cancer [103, 104]. The most commonly used agent is bevacizumab an anti-VEGF monoclonal antibody. Most of the clinical data is from the single-arm studies that show median progression-free survival of 2–4 months in the metastatic setting [105] and 2-year PFS of 60–70% in locally advanced tumors in combination with other therapeutic strategies [106–108]. A single randomized controlled study in locally advanced HNC showed it to be poor compared to standard of care [109].

Various tyrosine kinase inhibitors (TKI) have also been tried with limited success, and these sorafenib and sunitinib have been the main compounds tested in clinical trials in HNC [110]. Axitinib and pazopanib are also being tested in clinical trials in HNC [111]; however, so far, there is no recommendation for their regular use in guidelines.

## Conclusions

Hypoxia is a common factor in head and neck cancers and shows a cascade of response with intermediate biomarkers like HIF-1, HIF2, GLUT, and CA-9. The etiology of hypoxia appears to be multifactorial with angiogenesis and apoptosis playing an important role. Apart from HIF-1, VEGF appears to be an attractive therapeutic target; however, till date, there is no evidence of improvement in survival with the addition of these strategies to the standard of care. More basic studies looking into the mechanism of hypoxia and clinical trials exploring newer therapeutic compounds are needed to integrate hypoxia targeting in standard therapeutic strategies to treat HNC.

## Data Availability

Not applicable.

## Abbreviations

HNC: Head and neck cancer
HIF-1: Hypoxia-inducible factor-1
GLUT 1: Glucose transporter1
miRNAs: MicroRNAs
